# The Association of Type 2 Diabetes Mellitus with Cerebral Gray Matter Volume Is Independent of Retinal Vascular Architecture and Retinopathy

**DOI:** 10.1155/2016/6328953

**Published:** 2016-05-25

**Authors:** C. Moran, R. J. Tapp, A. D. Hughes, C. G. Magnussen, L. Blizzard, T. G. Phan, R. Beare, N. Witt, A. Venn, G. Münch, B. C. Amaratunge, V. Srikanth

**Affiliations:** ^1^Stroke and Ageing Research Group, Department of Medicine, School of Clinical Sciences at Monash Health, Monash University, Melbourne, VIC 3168, Australia; ^2^Department of Neurosciences, Monash Health, Melbourne, VIC 3168, Australia; ^3^Aged Care, Alfred Health, Melbourne, VIC 3162, Australia; ^4^Melbourne School of Population and Global Health, University of Melbourne, Parkville, VIC 3010, Australia; ^5^Singapore Eye Research Institute, Singapore National Eye Centre, Singapore 168751; ^6^International Centre for Circulatory Health, National Heart and Lung Institute, St Mary's Hospital and Imperial College, London SW7 2AZ, UK; ^7^Institute of Cardiovascular Science, University College London, London WC1E 6BT, UK; ^8^Menzies Institute for Medical Research, University of Tasmania, Hobart, TAS 7000, Australia; ^9^Research Centre of Applied and Preventative Cardiovascular Medicine, University of Turku, 20700 Turku, Finland; ^10^Developmental Imaging, Murdoch Children's Research Institute, Melbourne, VIC 7000, Australia; ^11^Department of Pharmacology and Molecular Medicine Research Group, School of Medicine, University of Western Sydney, Campbelltown, NSW 2753, Australia; ^12^Royal Victorian Eye and Ear Hospital, Melbourne, VIC 7000, Australia

## Abstract

It is uncertain whether small vessel disease underlies the relationship between Type 2 Diabetes Mellitus (T2DM) and brain atrophy. We aimed to study whether retinal vascular architecture, as a proxy for cerebral small vessel disease, may modify or mediate the associations of T2DM with brain volumes. In this cross-sectional study using Magnetic Resonance Imaging (MRI) scans and retinal photographs in 451 people with and without T2DM, we measured brain volumes, geometric measures of retinal vascular architecture, clinical retinopathy, and MRI cerebrovascular lesions. There were 270 people with (mean age 67.3 years) and 181 without T2DM (mean age 72.9 years). T2DM was associated with lower gray matter volume (*p* = 0.008). T2DM was associated with greater arteriolar diameter (*p* = 0.03) and optimality ratio (*p* = 0.04), but these associations were attenuated by adjustments for age and sex. Only optimality ratio was associated with lower gray matter volume (*p* = 0.03). The inclusion of retinal measures in regression models did not attenuate the association of T2DM with gray matter volume. The association of T2DM with lower gray matter volume was independent of retinal vascular architecture and clinical retinopathy. Retinal vascular measures or retinopathy may not be sufficiently sensitive to confirm a microvascular basis for T2DM-related brain atrophy.

## 1. Introduction

T2DM increases the risk of cognitive impairment and dementia [1]. Brain atrophy mediates a substantial portion of the association between T2DM and cognitive dysfunction [2]. Cerebrovascular disease, particularly disease of small cerebral vessels, has been postulated as a potential pathway leading to T2DM-related brain atrophy, possibly via inflammation and oxidative stress, advanced glycation, ischaemia, or infarcts [3, 4], but this is yet to be clarified. Although T2DM is commonly associated with microvascular disease [5], the associations between T2DM and brain MRI markers of small cerebral vessel disease such as white matter hyperintensities (WMH) or cerebral microbleeds are not strong, with contradictory findings in the literature [6]. The similar embryological, anatomical, and functional properties of retinal and cerebral vessels combined with ease of visualization of retinal vessels make them a potentially useful surrogate of cerebral small vessel disease [7, 8]. This has led to recent investigators using retinal vascular imaging measures in studies of people with and without cerebrovascular disease [7, 9–11], demonstrating their association with the risk of future stroke [12] and dementia [13–17]. T2DM is associated with features of clinical retinopathy [18] and changes in vessel architecture including greater retinal arteriolar diameter [19] and greater arteriolar tortuosity [20]. Therefore, it is possible that retinal vascular measures may assist in establishing a small vessel basis for T2DM-related brain atrophy.

To explore this issue further, we aimed to use retinal imaging to examine the roles of retinal vessel abnormalities and clinical retinopathy in explaining the association between T2DM and gray matter volume. We hypothesized that T2DM would be associated with measures of retinal vascular architecture and clinical retinopathy and that these measures would mediate or modify the association between T2DM and brain volume.

## 2. Materials and Methods

### 2.1. Sampling

We used a cross-sectional design. Our sampling methods have been described previously [2]. In brief, we recruited participants with T2DM and aged ≥55 years who were living in specific postcodes (7000–7199) of Southern Tasmania into the Cognition and Diabetes in Older Tasmanians (CDOT) study between January 2008 and January 2010. The National Diabetes Service Scheme (NDSS) was used as a sampling frame. The NDSS is administered by Diabetes Australia, providing products, information, and support to patients with T2DM who voluntarily enrol. The diagnosis of T2DM in the NDSS is based on physician assessment using standard criteria (fasting plasma glucose ≥7.0 mmol/L (126 mg/dL), random plasma glucose ≥11.1 mmol/L (199.8 mg/dL), or 2-hour glucose ≥11.1 mmol/L postoral glucose tolerance test), with registrants indicating willingness to participate in research. Exclusion criteria were people living in a nursing home signifying severe frailty, those with insufficient English for cognitive testing, contraindication to Magnetic Resonance Imaging (MRI), or presence of cataracts (to enable successful retinal imaging). We derived our comparison group from a sample of people aged ≥60 years without T2DM, recruited into the concurrently conducted population-based Tasmanian Study of Cognition and Gait (TASCOG). TASCOG participants were randomly identified from the electoral roll from the same postcodes as those in CDOT. Absence of T2DM in our comparison group was assessed using fasting plasma glucose <7.0 mmol/L, random plasma glucose <11.1 mmol/L, and Hb_A1c_ < 6.5% (48 mmol/mol) in those without a history of T2DM. Exclusion criteria were identical to the CDOT study. It must be noted that retinal vascular measurements were introduced into CDOT some months after commencement, resulting in a smaller overall sample size compared with our previously published paper describing brain atrophy in T2DM and its link with cognition [2]. The Southern Tasmanian Health and Medical Human Research Ethics Committee and the Monash University Human Research Ethics Committee approved the study and we obtained written informed consent.

### 2.2. Outcome Measurements

#### 2.2.1. Retinal Measurements

A 45° digital disc centred Canon CR-DGi nonmydriatic retinal camera was used to photograph two fields per eye. The digitised retinal images were graded by a single expert and processed using automated algorithms at the National Heart & Lung Institute, Imperial College, London, using well-established methods [21]. Color digital images (3072 × 2048 pixels) were converted to monochrome by extraction of the green layer. Using a custom written program in Matlab, vessel diameters were measured using a Sliding Linear Regression Filter (SLRF) [22] which achieves subpixel accuracy, and vessel length was measured between bifurcations using automatic tracking. SLRF is based on fitting a line by linear regression and progressively moving this across the entire section of interest, thus significantly decreasing noise. Measurements were made from ≥7 informative vessel segments and ≥5 bifurcations in both arterial and venous vessels from each subject. An informative vessel segment was defined to be either linking 2 clearly visible bifurcations or else traversing a linear distance of ≥1.5 disc diameters (the width of an average optic disc in photographic images) from the optic disc boundary without bifurcating. The analysis was performed on a sequence of complete trees generally from a single eye, continuing on the second eye if necessary to achieve the required number of vessel segments and bifurcations. The following measures of retinal vessel architecture were obtained: arteriolar and venular diameters and length; length/diameter ratios (LDR) of arteriolar segments (correct for refractive errors affecting measurements); arteriolar tortuosity; arteriolar bifurcation angles; arteriolar optimality ratio; and optimality deviance. Tortuosity was calculated as the ratio of arc length (*l*_*a*_) of the vessel segment (measured by tracking) to the straight line length of the segment (chord length, *l*_*c*_). The relationship of arteriolar diameters at bifurcations has been shown previously to relate to endothelial function [23]. Optimality ratio is the ratio of sum of “daughter” arteriolar diameters (*d*_1_) divided by the “parent” arteriolar diameter (*d*_0_) corrected for asymmetry [24]. Departure away from a theoretically predicted optimum [25] is an indicator of endothelial dysfunction [21]. Accuracy and reproducibility of our methods are high and have been previously described with SLRF repeatability coefficient of diameter measurement of 3.89 pixels [22].

Digital copies of the retinal photographs were also graded by a single clinical expert trained in ophthalmology (B. C. Amaratunge) for the presence/absence of features of any clinical retinopathy blinded to diabetes status. These included the presence of optic disc disease, copper/silver wiring, general arteriolar narrowing, focal arteriolar narrowing, arteriovenous crossing abnormalities, microaneurysm, intraretinal haemorrhage, nerve fibre haemorrhage, hard exudate, new vessel formation, cotton wool spots, macular degeneration, intraretinal microvascular abnormalities, and evidence of photocoagulation therapy. Retinopathy previously described as related to diabetes (diabetic retinopathy) was classified based on the features used in the simplified version of the Wisconsin grading system [26].

#### 2.2.2. Brain MRI

MRI scans were obtained using a single 1.5 T General Electric scanner with the following sequences: high-resolution T1-weighted spoiled gradient echo (SPGR) (TR 35 ms, TE 7 ms, flip angle 35°, field of view 24 cm, 120 contiguous slices, and isotropic voxel size 1 mm^3^); T2-weighted fast spin echo (TR 4300 ms; TE 120 ms; NEX 1; turbo factor 48; voxel size 0.90 × 0.90 × 3 mm); FLAIR (fluid attenuated inversion recovery) (TR = 8802 ms, TE = 130 ms, TI = 2200 ms, and voxel size 0.50 × 0.50 × 3 mm); gradient echo (GRE, TR = 0.8 ms; TE = 0.015; flip angle 30°; voxel size = 0.9 × 0.9 × 7 mm).

#### 2.2.3. Brain MRI Segmentation

3D-T1 and gradient echo (GRE) sequences were coregistered and a multispectral segmentation process [27] was used to produce tissue probability maps for gray matter and white matter using Statistical Parametric Mapping software version 8. Information from the GRE sequence was used to enhance contrast of subcortical structures prior to segmentation with SPM. Misclassifications of gray matter caused by white matter hyperintensities (WMH) were corrected using coregistered WMH maps produced using the automated procedure described previously [28]. Tissue probability maps in native space were used to compute tissue volume. Gray matter tissue maps were transformed to standard space for voxelwise regression using the nonlinear transformations estimated by the SPM tissue classification analysis. Images were modulated to preserve volume and smoothed with an isotropic Gaussian kernel, full width at half maximum (FWHM) = 8 mm. Voxel size of images in standard space was 1.5 mm^3^ voxels. A single expert manually segmented both hippocampi using established methods known to have high test-retest reliability in our laboratory (ICC 0.97) [29]. Using the tissue maps generated by these methods, total gray matter, white matter, and hippocampal volumes were calculated with voxel counting algorithms. WMH volumes were obtained by automated segmentation as described previously [2]. A single trained rater (C. Moran) determined the presence of MRI infarct and microbleed with confirmation by consensus between 2 stroke experts (T. G. Phan and V. Srikanth). Infarct was defined as a hypointensity ≥3 mm in diameter on 3D-T1-weighted and FLAIR images with a surrounding hyperintense rim on FLAIR [30]. Microbleeds were defined as small, rounded hypointense lesions with clear margins, ranging from 2 to 10 mm on GRE sequences. All measurements were blinded to group, age, sex, and outcome measures.

#### 2.2.4. Other Measurements

We used standardized questionnaires to record demographic and clinical information about duration of T2DM, years of formal education, health and medical history including vascular disease and risk factors, smoking, medication use, and alcohol use (g/day). We measured weight, height, and waist and hip circumferences and calculated Body Mass Index (BMI) as weight (kg)/height^2^ (m^2^); habitual physical activity by calculating mean number of steps per day using a Yamax pedometer worn over a seven-day period; mood using the 15-item Geriatric Depression Scale (GDS) [31]; and blood pressure (BP) using an Omron M4 sphygmomanometer in a sitting position as an average of 3 recordings from the right arm. Fasting plasma glucose was recorded using a Roche Cobas 6000 analyser with hexokinase determination and Hb_A1c_ using a Bio-Rad D10 analyser.

### 2.3. Data Analysis

Student's *t*-tests, Mann-Whitney *U* tests, and Chi square tests were applied as appropriate to compare mean scores and proportions of demographic and clinical variables between people with and without T2DM.

#### 2.3.1. Multivariable Regression

We first established the associations of T2DM with individual MRI measures adjusting in each regression for age and sex and additionally for total intracranial volume (except in the case of MRI infarcts and microbleeds). Linear regression was used for continuous outcome variables (total gray, total white, and right and left hippocampal volumes) and logistic regression for binary outcomes (infarct or microbleed). For all above analyses, potential for confounding was examined for additional covariates and adjustments made if addition of these terms changed the coefficient for T2DM by >10%. Covariates considered were hypertension (defined as mean BP > 140/90 mmHg or previous diagnosis), use of blood pressure lowering medications, hyperlipidaemia (yes/no), alcohol use (g/day), Hb_A1c_, ever smoked (yes/no), mean steps per day, ischemic heart disease, stroke, BMI, and waist-hip ratio.

We then examined the relationship between T2DM with the individual retinal measurements, before and after adjusting for age and sex and vascular risk factors. We also performed sensitivity analyses to examine whether measures of T2DM severity (Hb_A1c_ and duration of T2DM) or the use of blood pressure lowering medications altered the relationship between T2DM and the retinal measures. To examine whether retinal measures mediated the associations detected between T2DM and brain volume, we entered any retinal measure associated with T2DM into the model relating T2DM to brain volume measures adjusting for age, sex, and total intracranial volume. If the introduction of the retinal measure substantially attenuated the *β* coefficient for T2DM and the coefficient of the retinal measure remained unchanged from its unadjusted value without T2DM in the model, it was considered a potential mediator. We also examined for two-way interactions between T2DM and retinal measures using a test of significance of product terms. We applied standard regression diagnostics to assess the adequacy of models. Statistical analyses were carried out using STATA version 11.1 (StataCorp LP, College Station, TX).

Voxelwise regression was performed using SPM to examine relationships of optimality ratio and retinal vessel diameter with gray matter. Models were adjusted for total intracranial volume and tested with and without adjustment for age and sex. *p* values were corrected for multiple comparisons using familywise error rate of 0.05.

## 3. Results

Retinal photographs and MRI scans were available for 451 people, 270 with T2DM (mean age 67.3 years, SD 6.7), and 181 (mean age 72.9 years, SD 6.7) without T2DM. Sample characteristics and comparisons between those with and without T2DM are presented in Table 1. Those with T2DM reported median disease duration of 6 years (interquartile range 4–11 years), and 53 participants used insulin. Those with T2DM had greater fasting blood glucose and Hb_A1c_ levels, higher BMI and waist-hip ratio, and greater GDS score, were more likely to report a history of ischemic heart disease, hypertension, and hyperlipidaemia, and receive treatment with blood pressure lowering drugs and statins (all *p* < 0.05).

Retinal photographs could not be graded accurately in 15 people (10 with T2DM; 5 without T2DM) because of poor image quality. Those with nongradable retinal photographs were similar to those with gradable retinal photographs in age, sex, mean fasting blood glucose and Hb_A1c_ levels, BMI waist-hip ratio, GDS score, history of hypertension and hyperlipidaemia, and treatment with blood pressure lowering drugs and statins (data not shown). Compared with the sample described in our previously published report (*n* = 715) [2], the sample in this retinal substudy was smaller; participants were more likely to be on blood pressure reducing agents than those in the original sample (43% versus 61%, resp.) but were not different with respect to age, sex, and history of vascular risk factors (data not shown).

The comparisons of raw scores of retinal vascular measures are presented in Supplementary Tables 1 and 2 in Supplementary Material available online at http://dx.doi.org/10.1155/2016/6328953. The associations of T2DM with retinal measures are displayed in Table 2, first unadjusted, and followed by additional adjustment for age and sex and further for other covariates as required. Although T2DM was associated with larger arteriolar diameter (*p* = 0.03) and optimality ratio (*p* = 0.04) in univariable regression, these associations were attenuated by further adjustment. T2DM was not associated with any other measure of retinal vascular architecture. Among those with T2DM, there was no association between Hb_A1c_ or duration of T2DM and any of the retinal measurements. With respect to features of clinical retinopathy, a greater proportion of those with T2DM had copper/silver wiring, generalized and focal narrowing, arteriovenous crossing abnormalities, presence of microaneurysm, intraretinal haemorrhage, hard exudates, and macular degeneration (all *p* < 0.05). Overall there were no differences between groups in the proportions of people with the presence of retinopathy from any cause, but a significantly greater proportion with diabetic retinopathy in the T2DM group (12% versus 4%; *p* = 0.003) (Supplementary Table 2).

In the overall sample, only greater optimality ratio was associated with lower gray matter volume (mL) (*β* = −22.15 95% CI −41.69 to −2.61; *p* = 0.03) when adjusted for age, sex, and total intracranial volume. Adjusting for vascular risk factors only slightly weakened this rendering it not statistically significant (*β* = −19.13 95% CI −38.85 to 0.59; *p* = 0.06). Greater optimality ratio was also associated with a decreased risk of infarcts (*β* = −4.97 95% CI −9.62 to −0.33; *p* = 0.04). Adjusting for vascular risk factors weakened this association rendering it not statistically significant (*β* = −4.44 95% CI −9.41 to 0.53; *p* = 0.08). No associations were found between any measure of retinal architecture and MRI brain volumes. These associations did not differ in magnitude between those with and without T2DM, and there were no statistical interactions (Supplementary Table 3). Specifically, there were no associations between the presence of diabetic retinopathy and MRI measures (Supplementary Table 3). Voxelwise regression also did not reveal associations of optimality ratio or arteriolar diameter with gray matter.  Table 3 shows the effect of adding arteriolar diameter and optimality ratio to regression models of T2DM with brain MRI measures. Introduction of these two measures did not meaningfully change the association between T2DM and gray matter volume and other MRI measures in fully adjusted models. All the above described associations were not meaningfully different in those taking blood pressure lowering medications from those not taking blood pressure lowering medications.

## 4. Discussion

We examined the role of retinal vascular measures and clinical retinopathy in mediating the association between T2DM and MRI imaging biomarkers of brain disease. We found that T2DM was associated with increased retinal arteriolar diameter and optimality ratio and, as expected, with several measures of clinical retinopathy. Only greater retinal arteriolar optimality ratio was associated with lower gray matter volume. The association between T2DM and lower gray matter volume appeared to be independent of retinal vascular architecture and clinical retinopathy.

A number of studies have examined the association between retinal markers and brain imaging markers of cerebrovascular disease and neurodegeneration in older people [17, 32–35]. Some investigators have examined the relationship between retinal measures and cognitive function [15, 36], and one group has reported a cross-sectional association of retinopathy with lower gray matter volume in those with T2DM [37]. However, none have specifically examined whether retinal measures may mediate or modify the association between T2DM and brain volume by comparing those with and without T2DM. The relatively high prevalence of “any retinopathy” in our control group reflects the blinded rating of retinopathy. Although the prevalence of any retinopathy was similar between groups, the prevalence of diabetic retinopathy in our study (12%) was appropriately greater in those with T2DM and similar to that seen in other large community based studies of T2DM (~10%) [18, 38]. Measures of retinal vessel architecture have also been reported to be abnormal in T2DM probably as a result of chronic inflammation or endothelial dysfunction [39–41]. Consistent with some previous studies we found T2DM to be associated with larger retinal arteriolar diameter [19, 42] and for the first time demonstrate a relationship with optimality ratio, but not other measures of retinal architecture. It is possible that the lack of associations of T2DM with other retinal vascular measures could be explained by the close-to-target Hb_A1c_ (mean Hb_A1c_ 7.1%) and the high use of blood pressure lowering and cholesterol lowering medications in those with T2DM. However, controlling for Hb_A1c_, duration of diabetes, or the use of these medications did not significantly change the observed relationships. An alternative explanation is that retinal photography may not be sufficiently sensitive to measure disease at the level of true microvessels (blood retinal barrier) which is more likely to reflect the structure of the blood brain barrier or neurovascular unit. Dysfunction at the level of the neurovascular unit may still be a contributing factor for neuronal loss. Our results are further supported by a recent study in which Type 1 Diabetes Mellitus (T1DM) was associated with lower subcortical volume compared with those without T1DM and this association was independent of the presence of proliferative retinopathy [43]. Further work from the same group reported that those with T1DM and proliferative retinopathy had more cerebral microbleeds and skin capillary pathology, suggesting a generalized microangiopathy [44]. Those with T1DM often have greater disease duration and exposure to greater levels of hyperglycaemia than those with T2DM and therefore it is possible that duration and severity of T2DM are stronger predictors of vascular and cerebral complications than the diagnosis alone.

The mechanisms through which T2DM may contribute to lower gray matter volume may also include pathways that do not involve changes to microvascular architecture. T2DM is associated with chronic inflammation, oxidative stress [39–41], and, in a recent study, increased cerebrospinal levels of the protein tau [45], all of which can contribute to neuronal loss. Studies linking the pre-T2DM risk factors of obesity, insulin resistance, and the metabolic syndrome with brain atrophy further support the role of inflammation and oxidative stress as potential mediators of T2DM-related brain atrophy [46–48].

Strengths of our study include large sample size, robust phenotyping of T2DM, the use of fully automated brain segmentation, comprehensive measures of retinal vessel architecture and retinopathy, measurements blinded to diabetes status, and careful regression modelling to examine for mediation and effect modification. Importantly, our comparison sample was drawn from the same source population as those with T2DM. Our study has limitations. Participants on average were not very old and hence less likely to demonstrate substantial variation in MRI or retinal biomarkers. The sample in this study was smaller (*n* = 451) than that described in our previously published work (*n* = 713) [2]. This is because retinal photography was introduced as a measurement at a later stage of recruitment. Although recruitment strategies did not vary before and after introduction of retinal measures, it is possible that some selection bias may have been introduced that may have led to weaker associations being detected between T2DM and both retinal and MRI measurements. Although the two samples did not differ with respect to age, sex, and other vascular risk factors, the use of blood pressure lowering medication was higher in this study sample and this may have protected against more severe retinopathy or brain changes. However, the sample in this study was similar to our previously published work with regard to age, sex, and vascular risk factors. It is also possible that the retinal photographs of those with the most severe retinal changes may have been excluded due to poor quality, but those excluded did not appreciably differ from those included on other important characteristics. However, it remains possible that a greater load of retinal vascular disease may explain some of the T2DM-brain volume relationship. Given the cross-sectional design of our study, the results need to be confirmed in longitudinal analyses before firm conclusions can be drawn about the lack of a role for retinal vascular disease in explaining the T2DM-brain volume link. We examined a large number of associations between retinal measures (eleven) and structural brain measures (six). Multiple comparison testing increases the chance of finding an association where none exists and cannot be excluded as an explanation for the associations we found between optimality ratio and GMV.

## 5. Conclusions

The associations between T2DM and brain volume appeared independent of retinal measures. Longitudinal confirmation of these findings would be important in establishing pathways linking T2DM and dementia.

## Supplementary Material

Comparisons of continuous retinal variables between people with and without T2DM are presented in Supplementary Table 2, and comparisons of categorical retinal variables in Supplementary Table 3.

## Figures and Tables

**Table 1:** Sample characteristics.

	T2DM Mean (SD) or *n* (%) *n* = 270	No T2DM Mean (SD) or *n* (%) *n* = 181	*p* value
Age (years)	67.3 (6.7)	72.9 (6.7)	<0.001
Male sex	159 (59)	97 (54)	0.27
Formal education (years)	11.2 (3.6)	11.0 (3.9)	0.61
Systolic blood pressure (SBP) (mmHg)	135 (19)	137 (19)	0.23
Diastolic blood pressure (DBP) (mmHg)	76 (10)	78 (11)	0.01
Self-reported history of hypertension or mean SBP >140 or mean DBP >90 mmHg	223 (83)	123 (68)	<0.001
Use of blood pressure lowering medications	190 (70)	85 (47)	<0.001
Ischemic heart disease	57 (21)	27 (15)	0.01
TIA or stroke	22 (8)	9 (5)	0.19
Hyperlipidemia	127 (47)	10 (6)	<0.001
Statin use	163 (60)	45 (25)	0.005
Ever smoked	144 (53)	89 (49)	0.36
Alcohol intake (g/day)^*∗*^	12 (17)	17 (20)	0.001
BMI (kg/m^2^)	30.7 (5.1)	27.6 (4.3)	<0.001
Waist-hip ratio	0.96 (0.09)	0.90 (0.09)	<0.001
Median steps per day^*∗*^ (IQR)	5584 (3856–8461)	6328 (4471–8362)	0.21
GDS score^*∗*^	2.4 (2.6)	1.5 (1.6)	0.001
Gray matter volume (mL)	661.1 (64.4)	656.4 (63.7)	0.47
White matter volume (mL)	499.8 (53.2)	493.5 (49.3)	0.23
White matter hyperintensity volume (mL)	9.80 (6.3)	10.6 (7.1)	0.25
MRI infarct (presence)	54 (19)	22 (12)	0.03
MRI microbleed (presence)	9 (3)	12 (6)	0.79
Fasting blood glucose (mmol/L)	7.7 (2.1)	5.3 (0.6)	<0.001
Hb_A1c_ (%)	7.1 (1.2)	5.6 (0.3)	<0.001
Age at diabetes diagnosis^†^	57.6 (10.9)	NA	
Median duration of T2DM (years)^†^ (IQR)	6 (4–11)	NA	
Insulin use^†^	53 (20)	NA	

T2DM: Type 2 Diabetes Mellitus; TIA: Transient Ischemic Attack; BMI: Body Mass Index; GDS: Geriatric Depression Scale; MRI: Magnetic Resonance Imaging; SD: Standard Deviation; NA: not applicable; IQR: interquartile range; ^*∗*^Mann-Whitney *U* test; ^†^in those with T2DM.

**Table 2:** Association between Type 2 Diabetes Mellitus and retinal measurements^*∗*^.

Retinal variable	Model 1 *β* (95% CI)	Model 2 *β* (95% CI)	Model 3 *β* (95% CI)
*Arteriolar measures*			
Length, pixels	14.38 (−36.27 to 65.04)	13.01 (−42.00 to 68.01)	31.15 (−37.76 to 94.06)
Diameter, pixels	**0.48 (0.05 to **0.91)^†^	0.34 (−0.13 to 0.80)	0.01 (−0.52 to 0.54)
Length/diameter ratio	0.08 (−2.11 to 2.26)	0.12 (−2.24 to 2.49)	1.24 (−1.46 to 3.95)
Simple tortuosity	0.007 (−0.001 to 0.02)	0.006 (−0.003 to 0.01)	0.006 (−0.003 to 0.02)
Internal angle (°)	2.89 (−0.34 to 6.12)	2.83 (−0.66 to 6.33)	2.63 (−1.50 to 6.76)
Optimality ratio	**0.01 (0.001 to **0.02)^†^	0.01 (−0.01 to 0.02)	0.004 (−0.01 to 0.02)

*Venular measures*			
Length, pixels	3.87 (−31.40 to 39.14)	21.30 (−16.43 to 50.02)	8.30 (−35.99 to 52.59)
Diameter, pixels	0.08 (−0.61 to 0.77)	−0.21 (−0.95 to 0.54)	−0.45 (−1.32 to 0.43)
Length/diameter ratio	0.18 (−1.12 to 1.47)	0.95 (−0.44 to 2.33)	0.65 (−0.98 to 2.27)
Simple tortuosity	0.001 (−0.001 to 0.003)	0.002 (−0.001 to 0.004)	0.001 (−0.002 to 0.004)

*Retinopathy*			
Any retinopathy (odds ratio)	1.00 (0.68 to 1.47)	1.47 (0.96 to 2.26)	1.41 (0.85 to 2.32)

^*∗*^Including nonsymmetrical second-order vessels for all bifurcations.

Model 1 unadjusted.

Model 2 adjusted for age and sex.

Model 3 adjusted for age, sex, and vascular risk factors (history of smoking, hypertension, systolic and diastolic blood pressure, BMI and history of stroke, ischemic heart disease, and hyperlipidaemia).

^†^ *p* value < 0.05.

**Table 3:** Association between T2DM and MRI measures.

	Model 1 (T2DM)	Model 2 (*β* of T2DM)	Model 3 (*β* of T2DM)
Gray matter volume (mL)	**−3.60 (−6.28 to **−0.93)^**∗**^	**−3.70 (−6.39 to **−1.01)^**∗**^	**−3.68 (−6.36 to **−1.01)^**∗**^
Right hippocampal volume (mL)	0.07 (−0.006 to 0.15)	0.07 (−0.005 to 0.15)	0.07 (−0.005 to 0.15)
Left hippocampal volume (mL)	0.02 (−0.05 to 0.10)	0.02 (−0.05 to 0.10)	0.02 (−0.05 to 0.10)
Total hippocampal volume (mL)	0.08 (−0.05 to 0.22)	0.08 (−0.05 to 0.22)	0.08 (−0.05 to 0.22)
White matter volume (mL)	0.15 (−2.54 to 2.84)	0.24 (−2.47 to 2.94)	0.23 (−2.48 to 2.93)
White matter hyperintensity volume (mL)	−0.06 (−1.46 to 1.34)	−0.06 (−1.47 to 1.36)	−0.05 (−1.47 to 1.36)
Infarct present (odds ratio)	**2.31 (1.24 to **4.27)^**∗**^	**2.47 (1.32 to **4.63)^**∗**^	**2.55 (1.35 to **4.80)^**∗**^
Microbleed present (odds ratio)	0.47 (0.17 to 1.29)	0.50 (0.18 to 1.40)	0.50 (0.18 to 1.41)

T2DM: Type 2 Diabetes Mellitus; MRI: Magnetic Resonance Imaging.

Model 1, adjusted for age, sex, and intracranial volume.

Model 2 *β* of T2DM when adjusted for arteriolar diameter, age, sex, and intracranial volume.

Model 3 *β* of T2DM when adjusted for arteriolar diameter, optimality ratio, age, sex, and intracranial volume.

^**∗**^ *p* value < 0.01.
