# Supplementary material for: The Association of Type 2 Diabetes Mellitus with Cerebral Gray Matter Volume Is Independent of Retinal Vascular Architecture and Retinopathy
Source: J Diabetes Res. 2016 May 25;2016:6328953. doi: 10.1155/2016/6328953 (PMC4897713; doi:10.1155/2016/6328953)
Supplement: Supplementary file 1 — Comparisons of continuous retinal variables between people with and without T2DM are presented in Supplementary Table 2, and comparisons of categorical retinal variables in Supplementary Table 3. [file 6328953.f1.pdf]

Supplementary Table 1 Mean retinal measures*

	T2DM Mean (SD)	No T2DM Mean (SD)	P value
	270	181	
**Arteriolar measures**			
Arteriolar length, pixels	651 (264)	637 (274)	0.58
Arteriolar diameter, pixels	24.3 (2.3)	23.8 (2.2)	0.03
Length/diameter ratio	27.0 (11.3)	27.0 (12)	0.95
Simple tortuosity†	0.051 (0.04)	0.044 (0.04)	0.10
Internal Angle, °	75.5 (18.2)	72.6 (14.6)	0.08
Optimality ratio	0.83 (0.06)	0.82 (0.06)	0.04
**Venous Measures**			
Venous length, pixels	508 (180)	504 (202)	0.83
Venous diameter, pixels	29.4 (3.7)	29.3 (3.7)	0.81
Venous length/diameter ratio	17.7 (6.8)	17.5 (7.2)	0.79
Venous simple tortuosity†	0.021 (0.01)	0.020 (0.01)	0.24

T2DM: Type 2 diabetes; SD: Standard Deviation

*Including non-symmetrical second order vessels for all bifurcations.

† Mann-Whitney U test

Supplementary Table 2. Descriptive characteristics of retinopathy changes

	T2DM N(%) n=283	No T2DM N(%) n=187	p value
Disc disease	8 (3)	11 (6)	0.10
Copper/silver wiring	47 (17)	16 (9)	0.01
Generalised narrowing	31 (11)	34 (18)	0.03
Focal narrowing	4 (1)	13 (7)	0.002
A-V crossing	17 (6)	24 (13)	0.01
Microaneurysm *	22 (8)	1 (1)	<0.001
Venous diameter change	5 (2)	1 (1)	0.24
Intraretinal haemorrhage	25 (9)	2 (1)	<0.001
Nerve fibre haemorrhage *	10 (4)	3 (2)	0.21
Hard exudate	10 (4)	0	0.009
New vessel formation *	0	0	
IRMA[a]	0	0	
Cotton wool spot *	3 (1)	2 (2)	0.99
Macular degeneration	8 (3)	16 (9)	0.006
Photocoagulation Treatment *	11 (4)	0	0.006
Diabetic retinopathy *	33 (12)	7 (4)	0.003
Any retinopathy	106 (37)	70 (37)	1.0

A-V- Arteriovenous crossing; IRMA – intraretinal microvascular abnormalities; NA – not applicable

* Retinopathy changes used to classify diabetes-related retinopathy using a simplified version of the Wisconsin grading system (25)

Supplementary Table 3 Associations between retinal measurements and brain measurements

Retinal variable	Gray matter volume (ml) β (95% CI)	Total hippocampal volume (ml) β (95% CI)	White matter volume (ml) β (95% CI)	WMH volume (ml) β (95% CI)	Infarct present* β (95% CI)	Microbleed present* β (95% CI)
**Arteriolar measures**						
Length						
WG	0.002(-0.003-0.006)	-0.0002(-0.0004-0.00001)	0.002(-0.002 to 0.006)	0.0002(-0.002 to 0.003)	-0.0003 (-0.001 to 0.001)	-0.001(-0.003 to 0.001)
T2DM	0.002 (-0.003-0.008)	-0.0001(-0.0004-0.0002)	0.004 (-0.002-0.01)	0.0005(-0.002-0.003)	-0.001(-0.002-0.0004)	-0.002(-0.005-0.001)
No T2DM	0.002 (-0.005-0.080)	-0.0002(-0.001-0.0001)	-0.001 (-0.01 -0.01)	-0.0003(-0.004-0.004)	0.0004(-0.001-0.002)	-0.001(-0.003-0.002)
Diameter						
WG	0.10(-0.42 to 0.62)	0.004 (-0.03 to 0.03)	-0.22(-0.74 to 0.31)	0.006(-0.27 to 0.28)	-0.07(-0.19 to 0.05)	-0.12(-0.33 to 0.10)
T2DM	0.49 (-0.18 to 1.17)	0.005 (-0.03 to 0.04)	-0.20 (-0.88 to 0.47)	-0.06 (-0.39 to 0.26)	-0.10 (-0.24 to 0.05)	-0.30 (-0.65 to 0.05)
No T2DM	-0.31 (-1.12 to 0.51)	-0.005 (-0.04 to 0.04)	-0.26 (-1.11 to 0.58)	0.11 (-0.38 to 0.61)	-0.10 (-0.31 to 0.12)	0.05 (-0.23 to 0.33)
LDR						
WG	0.04(-0.06 to 0.14)	-0.004 (-0.01 to 0.001)	0.05(-0.05 to 0.16)	-0.003(-0.06 to 0.05)	-0.01(-0.03 to 0.02)	-0.02(-0.06 to 0.02)
T2DM	0.04 (-0.09 to 0.18)	-0.004 (-0.01 to 0.003)	0.09 (-0.04 to 0.22)	0.005 (-0.06 to 0.07)	-0.02 (-0.04 to 0.01)	-0.02 (-0.09 to 0.05)
No T2DM	0.04 (-0.11 to 0.20)	-0.005 (-0.01 to 0.003)	-0.005 (-0.17 to 0.16)	-0.02 (-0.11 to 0.08)	0.01 (-0.03 to 0.05)	-0.02 (-0.07 to 0.04)
Simple tortuosity WG	4.28(-24.77 to 33.32)	-0.58 (-2.06 to 0.89)	2.75(-26.29 to 31.80)	1.55(-13.61 to 16.70)	-1.04(-7.49 to 5.42)	2.42(-8.53 to 13.37)
T2DM	3.39 (-32.73 to 39.51)	-1.12 (-3.00 to 0.76)	6.16 (-29.92 to 42.24)	-0.07 (-17.59 to 17.44)	-1.26 (-9.00 to 6.47)	1.65 (-14.72 to 18.02)
No T2DM	17.33 (-32.06 to 66.71)	-0.04 (-2.48 to 2.41)	-15.51 (-66.14 to 35.12)	1.86 (-27.83 to 31.54)	-1.14 (-14.21 to 11.94)	5.88 (-9.54 to 21.29)
Internal Angle						
WG	-0.05(-0.12 to 0.02)	-0.0001 (-0.004 to 0.003)	0.05(-0.02 to 0.12)	-0.01(-0.05 to 0.02)	0.003(-0.01 to 0.02)	-0.02(-0.05 to 0.01)
T2DM	-0.05 (-0.13 to 0.03)	-0.001 (-0.006 to 0.003)	0.06 (-0.02 to 0.14)	-0.01 (-0.05 to 0.03)	0.002 (-0.01 to 0.02)	-0.01 (-0.05 to 0.03)
No T2DM	-0.03 (-0.16 to 0.11)	0.002 (-0.004 to 0.008)	-0.01 (-0.15 to 0.13)	-0.01 (-0.09 to 0.07)	-0.004 (-0.04 to 0.03)	-0.02 (-0.07 to 0.03)
Optimality Ratio						
WG	**-22.15(-41.69- -2.61)**†	-0.54(-1.52 to 0.45)	13.97(-5.63 to 33.56)	3.70(-13.95 to 6.54)	**-4.97(-9.62 to -0.33)**†	-5.52(-14.10 to 3.07)
T2DM	-17.63 (-43.87 to 8.61)	-0.03 (-1.38 to 1.33)	7.75 (-18.25 to 33.76)	-0.88 (-13.48 to 11.71)	**-6.57 (-12.56 to -0.58)**†	-5.31 (-17.62 to 7.00)
No T2DM	-26.00 (-54.94 to 2.95)	-1.30 (-2.71 to 0.10)	20.86 (-9.10 to 50.83)	-7.77 (-25.41 to 9.87)	-3.79 (-11.95 to 4.38)	-5.21 (-17.07 to 6.65)

**Venular measures**						
Length<br>WG<br>T2DM<br>No T2DM	-0.001(-0.008-0.005)<br>0.001 (-0.01 to 0.01)<br>-0.003 (-0.01 to 0.01)	-0.0001(-0.0004 to 0.0002)<br>-0.0002 (-0.001 to 0.0003)<br>-0.00004(-0.0005 to 0.0004)	-0.006(-0.01 to 0.0001)<br>-0.006 (-0.01 to 0.002)<br>-0.005 (-0.01 to 0.005)	0.001(-0.002 to 0.004)<br>-0.0005 (-0.005 to 0.004)<br>0.002 (-0.003 to 0.008)	0.0003(-0.001 to 0.002)<br>0.001 (-0.001 to 0.002)<br>-0.001 (-0.003 to 0.002)	-0.002(-0.005 to 0.001)<br>0.001 (-0.003 to 0.004)<br>-0.004 (-0.01 to 0.001)
Diameter<br>WG<br>T2DM<br>No T2DM	0.29(-0.03 to 0.60)<br>0.36 (-0.04 to 0.76)<br>0.10 (-0.40 to 0.61)	0.001(-0.01 to 0.02)<br>-0.001 (-0.02 to 0.02)<br>0.01 (-0.02 to 0.03)	0.004(-0.31 to 0.32)<br>-0.10 (-0.50 to 0.30)<br>0.20 (-0.32 to 0.72)	-0.04(-0.21 to 0.12)<br>-0.16 (-0.35 to 0.04)<br>0.15 (-0.15 to 0.45)	0.96 (0.89 to 1.03)<br>0.95 (0.88 to 1.04)<br>0.96 (0.84 to 1.10)	1.02(0.90 to 1.15)<br>0.99 (0.82 to 1.19)<br>1.03 (0.88 to 1.21)
LDR<br>WG<br>T2DM<br>No T2DM	-0.08(-0.25 to 0.09)<br>-0.04 (-0.26 to 0.18)<br>-0.08 (-0.34 to 0.18)	-0.003(-0.01 to 0.006)<br>-0.005 (-0.02 to 0.01)<br>-0.002 (-0.02 to 0.01)	-0.13(-0.30 to 0.04)<br>-0.11 (-0.33 to 0.11)<br>-0.18 (-0.44 to 0.09)	0.03(-0.06 to 0.12)<br>0.03 (-0.08 to 0.14)<br>0.03 (-0.12 to 0.18)	0.02(-0.02 to 0.05)<br>0.03 (-0.01 to 0.07)<br>-0.02 (-0.09 to 0.05)	-0.04(-0.12 to 0.03)<br>0.02 (-0.07 to 0.11)<br>-0.11 (-0.24 to 0.01)
Simple tortuosity<br>WG<br>T2DM<br>No T2DM	-25.58(-114.88 to 63.72)<br>18.58 (-98.88 to 136.0)<br>-58.92(-197.13to 79.3)	-0.53(-5.14 to 4.08)<br>-1.63 (-7.87 to 4.61)<br>-0.15 (-7.07 to 6.77)	-51.25(-140.99 to 38.49)<br>-26.16 (-143.66 to 91.34)<br>-106.9 (-248.80 to 35.00)	19.73(-28.64 to 64.51)<br>37.00 (-20.10 to 94.10)<br>-13.00 (-94.69 to 68.71)	8.98(-10.32 to 28.28)<br>12.92 (-11.31 to 37.15)<br>-4.47 (-43.23 to 34.29)	-23.50(-66.46 to 19.45)<br>-1.93 (-56.08 to 52.23)<br>-50.56 (-129.74 to 28.62)
Diabetic retinopathy<br>T2DM	0.30 (-4.44 to 5.04)	0.25 (-0.22 to 0.27)	-3.00 (-7.55 to 1.54)	-0.14 (-2.54 to 2.26)	0.43 (-0.48 to 1.34)	0.65 (-1.00 to 2.30)

LDR: length/diameter ratio; WG: whole group combined; T2DM: Type 2 diabetes; WMH: White matter hyperintensity

Model 1, adjusted for age, sex and intra-cranial volume

* Not adjusted for history of stroke or intra-cranial volume

† p value < 0.05
